# Different Immunity Elicited by Recombinant H5N1 Hemagglutinin Proteins Containing Pauci-Mannose, High-Mannose, or Complex Type N-Glycans

**DOI:** 10.1371/journal.pone.0066719

**Published:** 2013-06-14

**Authors:** Shih-Chang Lin, Jia-Tsrong Jan, Ben Dionne, Michael Butler, Ming-Hsi Huang, Chung-Yi Wu, Chi-Huey Wong, Suh-Chin Wu

**Affiliations:** 1 Institute of Biotechnology, National Tsing Hua University, Hsinchu, Taiwan; 2 Genomics Research Center, Academia Sinica, Taipei, Taiwan; 3 Department of Microbiology, University of Manitoba, Winnipeg, Manitoba, Canada; 4 National Institute of Infectious Diseases and Vaccinology, National Health Research Institutes, Zhunan, Taiwan; McGill University, Canada

## Abstract

Highly pathogenic avian influenza H5N1 viruses can result in poultry and occasionally in human mortality. A safe and effective H5N1 vaccine is urgently needed to reduce the pandemic potential. Hemagglutinin (HA), a major envelope protein accounting for approximately 80% of spikes in influenza virus, is often used as a major antigen for subunit vaccine development. In this study, we conducted a systematic study of the immune response against influenza virus infection following immunization with recombinant HA proteins expressed in insect (Sf9) cells, insect cells that contain exogenous genes for elaborating N-linked glycans (Mimic) and mammalian cells (CHO). While the antibody titers are higher with the insect cell derived HA proteins, the neutralization and HA inhibition titers are much higher with the mammalian cell produced HA proteins. Recombinant HA proteins containing tri- or tetra-antennary complex, terminally sialylated and asialyated-galactose type N-glycans induced better protective immunity in mice to lethal challenge. The results are highly relevant to issues that should be considered in the production of fragment vaccines.

## Introduction

Highly pathogenic avian influenza (HPAI) viruses such as H5N1, H7N7, and H9N2 can result in poultry and occasionally in human mortality [1]. The first instance of human HPAI H5N1 virus infection occurred in Hong Kong in 1997; it re-emerged in 2003 and has triggered sporadic human infections in Asia, the Middle East, Europe, and Africa with a mortality rate that could be as high as 60% [2] but the true mortality rate of H5N1 infected individuals is currently unknown [3]. Humans can be infected with H5N1 from close contact with infected poultry, and virus mutations have been identified in cases of cross-human transmission. Recent reports indicate that the involvement of HA and PB2 amino acid substitutions leads to easier transmission in ferrets, suggesting that HPAI H5N1 viruses have the potential to evolve and be transmitted between mammals, thus posing the risk of a human pandemic [4], [5]. Accordingly, an effective H5N1 vaccine is urgently needed to reduce pandemic potential.

HA, a major envelope protein accounting for approximately 80% of spikes in influenza virions, is often used as a major antigen for subunit vaccine development. Anti-H5N1 neutralizing antibodies have been elicited in mice, chickens, and ferrets using recombinant HA proteins expressed in mammalian and insect cells [6]–[8], plant cells [9], [10], and E. coli cells [11]–[15]. Recombinant HA proteins from mammalian and insect cells are capable of more authentic post-translational modifications (e.g., disulfide bond formation and complex type glycosylation) that facilitate protein folding and stability [16]. Complex N-linked HA glycoproteins expressed in mammalian cells have been described as eliciting stronger immune responses compared to pauci-mannose N-glycans expressed in insect cells [7], [8]. At least two research teams have reported that single GlcNAc glycans of complex N-linked HA glycoproteins increase receptor binding in sialic acid and neutralizing antibody titers in mice [7], [17].

To investigate the immunogenicity of HA bearing different N-glycans, we created four recombinant HA proteins using one mammalian (CHO) and two insect (Sf9 and Mimic) cell lines with or without neuraminidase (NA) treatment. Results show that the recombinant HA proteins carrying pauci-mannose and high-mannose glycans elicited higher titers of HA-specific IgG antibodies and stronger T cell responses compared to recombinant HA proteins carrying complex-type glycans. Recombinant HA proteins carrying tri- and tetra-antennary complex-type glycans induced even higher neutralizing and hemagglutinin-inhibiting (HI) antibody titers, thus enhancing protective immunity. The results are highly relevant to issues that should be considered in the production of fragment vaccines.

## Results

### Recombinant HA protein expression and characterizing N-linked glycans

**Figure 1:**
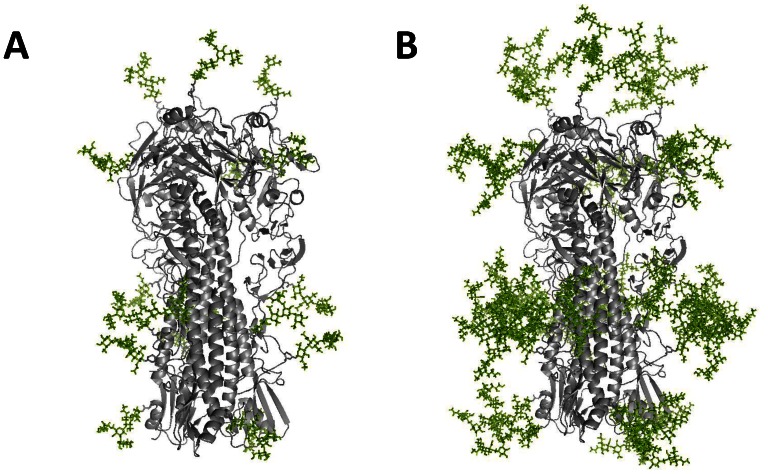
3D structure model. (A) Insect cell expressed HA attached with pauci-mannose N-glycans and (B) mammalian cell expressed HA attached with complex-type N-glycans were created by the crystal structure of HA (A/Vietnam/1194/04, PDB ID: 2IBX) and Glyprot.

**Figure 2:**
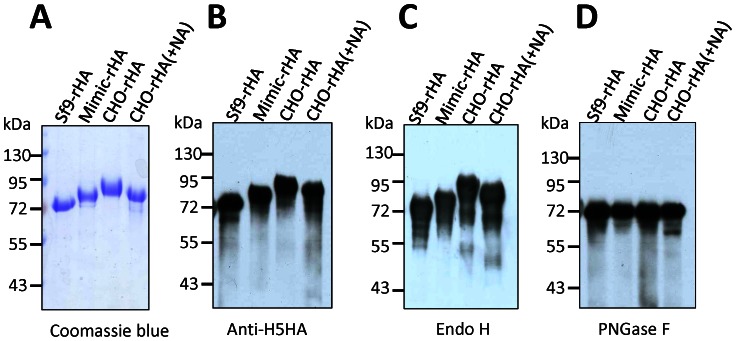
Recombinant HA protein expression and purification . Soluble HA proteins were constructed using the HA cDNA sequences of H5N1 A/Thailand/1(KAN-1)/2004. The PQRERRRKKRG multibasic protease cleavage site between HA_1_ and HA_2_ was mutated to PQRETRG to retain the uncleaved protein. The C-terminal sequence was fused with a GCN4pII leucine zipper sequence and ended with a His-tag to facilitate purification. Recombinant H5HA proteins were purified from the culture supernatants of Sf9, Mimic, and CHO cells and analyzed by SDS-PAGE with (A) Coomassie Blue staining and (B) western blotting with anti-H5 antibodies. Proteins were also analyzed following treatment with (C) Endo H and (D) PNGase F.

The 3D protein structures of the pauci-mannose and complex-type N-glycans attached to the trimeric H5N1 influenza HA protein (A/Vietnam/1194/04) were created using the crystal structure of HA from A/Vietnam/1194/04 strain (PDB ID: 2IBX) and Glyprot [18]. These structures clearly elaborate the differences between insect cell expressed HA (**Figure 1A**) and mammalian cell expressed HA (**Figure 1B**). For insect cell expression, the soluble recombinant HA-expressing coding sequences were cloned into a pFast-Bac vector to obtain recombinant baculoviruses for infecting Sf9 and Mimic cells. For CHO cell (mammalian) expression, the HA coding sequence was optimized and cloned into a pISID expression vector containing intron splicing; IRES-driven *dhfr* gene amplification was performed as described in Lin et al. (2010) [19]. Recombinant HA proteins were obtained from the culture supernatants of Sf9, Mimic, and CHO cells and purified using nickel-chelated affinity chromatography. Results from the Coomassie blue staining of SDS-PAGE gels and anti-H5HA antibodies indicate that recombinant HA proteins from Sf9 cells (Sf9-rHA) had lower molecular weights than the HA proteins from Mimic cells (Mimic-rHA), and that the recombinant HA proteins from CHO cells (CHO-rHA) had the highest molecular weights (**Figure 2A,B**). Purified recombinant proteins of sf9-rHA, Mimic-rHA, and CHO-rHA obtained from nickel-chelated affinity chromatography were >90% as shown by Coomassie blue staining (**Figure 2A**). Although the purified HA protein was clearly the major component of the final preparations, host cell contaminants (i.e. DNA) and microbial or endotoxin contaminants were also present by the lab-scale purification equipment and procedures. However, the levels of DNA and endotoxin are relatively minor to affect immune responses in this study. We only detected low-level endotoxin in the purified recombinant HA proteins since cultured insect and mammalian cells were used for HA expression (data not shown).

To further mimic the HA structures in vivo where the viral neuraminidase (NA) is active, CHO-rHA proteins were treated with excessive bacterial NA, CHO-rHA(+NA), to remove terminal sialic acid. Figures 2A and 2B demonstrate the removal of terminal sialic acid with decreased molecular weights (CHO-rHA vs. CHO-rHA(+NA)). To further confirm that changing molecular weights reflected differences in glycan form, recombinant HA proteins were treated with Endo H and PNGase F. The high-to-low order of molecular weights following treatment with Endo H was CHO-rHA > CHO-rHA(+NA) > Mimic-rHA > Sf9-rHA (**Figure 2C**). Since Endo H only cleaves the mannose-terminated N-glycans, no molecular weight shifts were observed for CHO-rHA and CHO-rHA(+NA) (**Figure 2C** vs. **Figure 2A,B**). In contrast, all four recombinant HA proteins had approximately equal molecular weights following treatment with PNGase F (**Figure 2D**). The results confirm that different N-linked glycans were added to the HA glycoproteins via insect or mammalian cell expression. Since similar molecular weights were observed in sf9-rHA and CHO-rHA after PNGase F treatment (**Figure 2D**), the alpha 1-3 fucose on the first GlcNAc, which was resistant to PNGase F, was not the major glycan structure in sf9-rHA. However, no alpha 1-3 fucose exists in CHO-rHA since CHO cells only introduce alpha 1,6-fucose to N-glycans [**20**].

**Figure 3:**
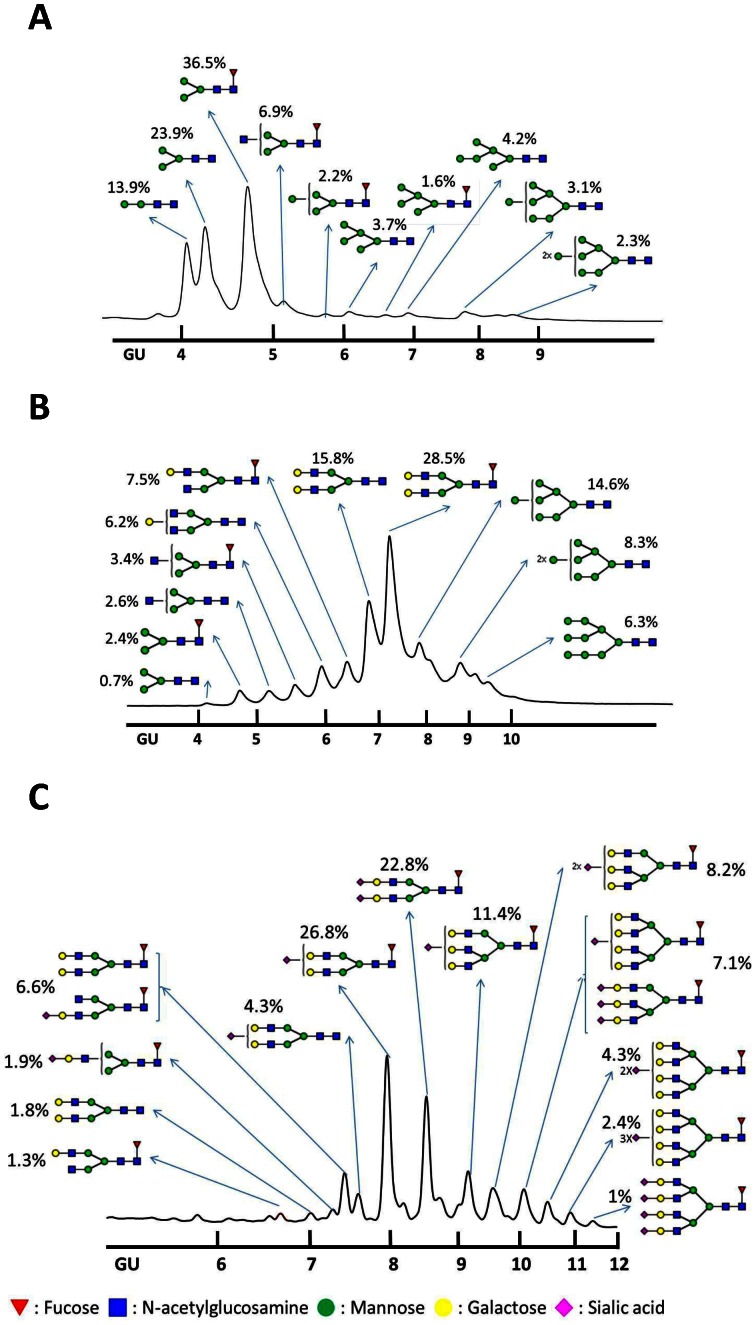
HILIC-HPLC glycan patterns. Purified recombinant HA proteins were placed on SDS-PAGE gels and deglycosylated using PNGase F. Glycans were isolated from protein backbones and analyzed using HILIC-HPLC to identify individual glycan structures. A dextran ladder was created with HILIC-HPLC and used as a standard to provide glucose unit (GU) values for individual peaks recognized in glycan samples from recombinant HA proteins. Shown are HA N-glycan profiles of (A) Sf9-rHA, (B) Mimic-rHA, and (C) CHO-rHA. Red inverted triangles, fucose; blue squares, N -acetylglucosamine; green circles, mannose; yellow circles, galactose; pink diamonds, sialic acid.

Sf9-rHA, Mimic-rHA, and CHO-rHA N-linked glycan patterns were further analyzed using hydrophilic interaction liquid chromatography- high-performance liquid chromatography (HILIC-HPLC) [21], which has been performed to analyze the glycoforms of recombinant human β-interferon [22], [23] and recombinant human erythropoietin [24]. The Sf9-rHA primarily contained pauci-mannose (Man_2-3_GlcNAc_2_+/−Fuc; 74.3%) and high-mannose glycans (Man_4-9_GlcNAc_2_+/−Fuc; 17.1%), along with a much smaller amount of hybrid glycans (Man_3_GlcNAc_3_Fuc; 6.9%) (**Figure 3A**). The Mimic-rHA primarily contained bi-antennary complex glycans without terminal sialic acid (Gal_1-2_Man_3_GlcNAc_4_+/−Fuc; 58%), along with high-mannose (Man_7-9_GlcNAc_2_+/−Fuc; 29.2%), hybrid (Man_3_GlcNAc_3_+/−Fuc; 6%), and minor pauci-mannose glycans (Man_3_GlcNAc_2_+/−Fuc; 3.1%) (**Figure 3B**). Since Mimic cells were derived from Sf9 cells and integrated with mammalian glycosyltransferases [25], [26], Mimic-rHA mostly contained high-mannose or complex glycans with bi-antennary structures (**Figure 3B**). No sialylation was observed and tri-antennary structures were not extended on rHA from the Mimic cells. CHO-rHA mostly contained complex glycans with bi-, tri, and tetra-antennary structures containing one or more terminal sialic acids (Sia_1-4_Gal_1-4_Man_3_GlcNAc_4-6_+/−Fuc; 94.9%) (**Figure 3C**). The CHO-rHA(+NA) glycan patterns consisted of complex N-glycans with bi-, tri-, and tetra-antennary structures terminated at galactose ends due to sialic acid-associated NA cleavage. Mimic-rHA, CHO-rHA, and CHO-rHA(+NA) only contained partially mannose-terminated glycans, which could be cleaved by Endo-H, confirming that the molecular weight shift in SDS-PAGE was not clearly observed after Endo H treatment (**Figure 2C**).

### HA-specific IgG titer and IgG-binding avidity

**Figure 4:**
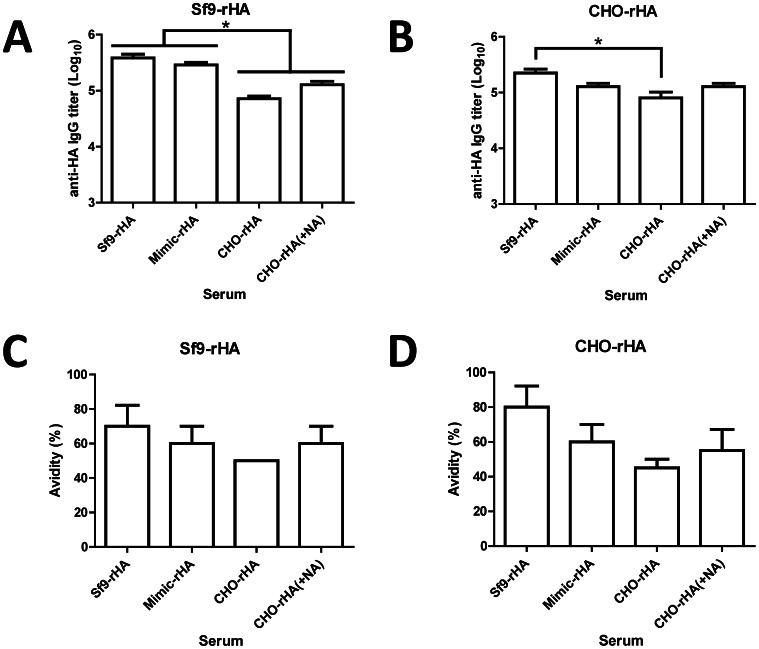
Antibody responses elicited by recombinant HA proteins in immunized mice. Female BALB/c mice (6-8 wk old, five mice per group) were intramuscularly immunized with 20 µg of recombinant HA proteins over a 3-week interval; blood was collected 2 wk after the second inoculation. Total anti-HA IgG antibody titers were measured using ELISAs with (A) Sf9-rHA or (B) CHO-rHA. Total IgG antibody avidities were measured after treatment with 6 M urea followed by ELISA binding to (C) Sf9-rHA or (D) CHO-rHA. Data represent geometric mean ± standard error; results were analyzed using one-way ANOVAs with Tukey's tests, with statistical significance at *p*<0.05 as asterisks indicated.

Group of BALB/c mice (5 mice per group) were immunized at three-week intervals with two 20 µg doses of Sf9-rHA, Mimic-rHA, CHO-rHA, or CHO-rHA(+NA) proteins, all containing the PELC+CpG adjuvant [27], [28]. We only investigated the immune responses elicited by recombinant HA proteins coupled with PELC+CpG adjuvant. Although it is unlikely that the non-adjuvanted recombiant HA proteins can elicit detectable neutralizing antibody response, we still cannot conclude the antigen glycoforms may elicit different B and T cell responses without adjuvant. Antisera were collected 2 wk following the second inoculations. Total anti-HA IgG antibody titers were measured using ELISA (Sf9-rHA, **Figure 4A**; CHO-rHA, **Figure 4B**). Our results indicate that Sf9-rHA immunization elicited the highest anti-HA total IgG titers, and CHO-rHA immunization the lowest among these four groups investigated.

Antibody avidity is the strength of total bond interactions between antibody and antigen. Avidity ELISA is a quantitative test to determine the strength between antibody and antigen. Low avidity antibodies are eluted after urea, a mild protein denaturing agent, treatment. A ratio is derived by comparing the antibody titers in the presence and absence of urea, and high avidity antibodies will have a higher ratio than low avidity antibodies [29−31]. To confirm these differences, we measured anti-HA IgG antibody avidity titers using 6 M urea with ELISA plates coated with Sf9-rHA (**Figure 4C**) or CHO-rHA (**Figure 4D**). Again, the results indicate that Sf9-rHA had the highest avidity and CHO-rHA the lowest among these four groups investigated.

### Distribution of HA-specific IgM, IgA and IgG subtype isotypes and antibody-secreting B cells in splenocytes

**Figure 5:**
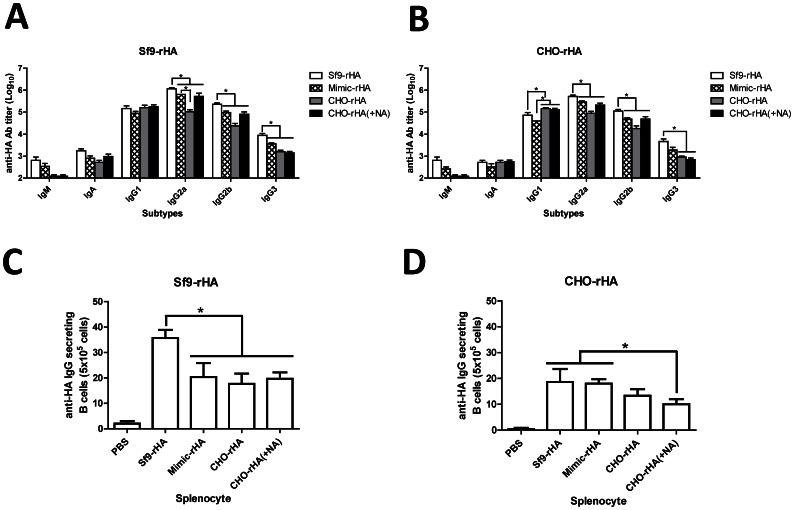
Antibody isotypes/subtypes and antibody-secreting B cells. The antibody isotypes of IgM and IgA and the IgG1, IgG2a, IgG2b and IgG3 subtypes in sera were analyzed using ELISA plates coated with (A) Sf9-rHA or (B) CHO-rHA. Mouse splenocytes were collected 3 wk following second inoculations, and then reacted with (C) Sf9-rHA or (D) CHO-rHA to measure the numbers of antibody-secreting B cells in spleens; ELISPOT assays were performed using HRP-conjugated anti-mouse IgG antibodies. Data represent mean ± standard deviation; results were analyzed using one-way ANOVAs with Tukey's tests, with statistical significance at *p*<0.05 as asterisks indicated.

To confirm the affinity maturation levels triggered by different glycan HA proteins, we measured the relative levels of IgM, IgA and IgG subtypes in sera immunized with Sf9-rHA, Mimic-rHA, CHO-rHA, and CHO-rHA(+NA) using ELISAs coated with Sf9-rHA (**Figure 5A**) or CHO-rHA (**Figure 5B**). Sf9-rHA and Mimic-rHA immunizations elicited slightly higher levels of IgM compared to the CHO-rHA and CHO-rHA(+NA) immunizations; no significant differences were found for serum IgA levels. However, different profiles emerged from IgG1, IgG2a, IgG2b and IgG3 distributions. For IgG1, CHO-rHA and CHO-rHA(+NA) immunizations resulted in higher titers compared to Sf9-rHA and Mimic-rHA immunization when ELISA plates were coated with CHO-rHA. For IgG2a, IgG2b, and IgG3, Sf9-rHA immunization elicited higher levels compared to Mimic-rHA, CHO-rHA and CHO-rHA(+NA) immunization when ELISA plates were coated with Sf9-rHA or CHO-rHA. Accordingly, HA containing pauci-mannose type N-glycans (Sf9-rHA) induced higher levels of the IgG2a, IgG2b and IgG3 subtypes. In contrast, HA containing the complex type N-glycans CHO-rHA and CHO-rHA(+NA) elicited higher IgG1 subtype antibodies.

Splenocytes collected from each group of mice 3 wk following the second inoculations were reacted with Sf9-rHA (**Figure 5C**) and CHO-rHA (**Figure 5D**) and examined using ELISPOT assays to measure quantities of HA-specific antibody-secreting B cells in spleens. The numbers elicited by Sf9-rHA were significantly higher than those for the other three immunization groups, and larger foci were found in antibody-secreting B cells detected by Sf9-rHA compared to those detected by CHO-rHA in all four immunization groups.

### T cells in splenocytes and T cell stimulation by antigen-presenting dendritic cells

**Figure 6:**
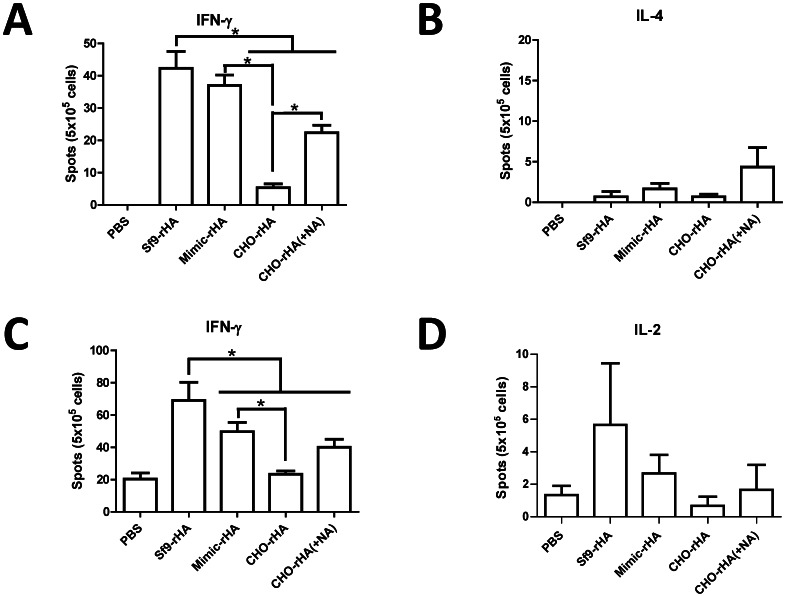
T cell responses in splenocytes and T-cell stimulation by antigen-presenting dendritic cells. Splenocytes were added to each well in 96-well plates (5×10^5^ cells/well) and stimulated with 1 µg/ml pooled peptides (15-mer overlapping 8 amino acids) spanning the HA_1_ of H5HA (A/Viet Nam/1203/2004) to determine (A) IFN-γ- and (B) IL-4-secreting T cells using ELISPOT assays. Bone marrow-derived dendritic cells (DCs) were treated with LPS and recombinant HA proteins and then fixed with paraformaldehyde followed by quenching with glycine. Pre-treated DCs were co-incubated with splenocytes from mice immunized with Sf9-rHA for 2 d. Antigen presentation was determined by measuring (C) IFN-γ- and (D) IL-2-secreting T cells using ELISPOT assays. DCs pretreated with LPS and pulsed with PBS were used as a negative control. Data represent mean ± standard deviation. Results were analyzed using two-tailed Student’s *t* tests with statistical significance at *p*<0.05 as asterisks indicated.

Splenocytes collected from each group of mice 3 wk following their second inoculations were stimulated with H5HA_1_ peptide pool and tested using ELISPOT assays to determine quantities of IFN-γ- and IL-4-secreting T cells. Our results indicate a high-to-low order of IFN-γ-secreting T cells in the four immunization groups as Sf9-rHA > Mimic-rHA > CHO-rHA(+NA) > CHO-rHA (**Figure 6A**). For IL-4-secreting T cells, no significant differences were observed among the four immunization groups (**Figure 6B**).

For T cell recognition, antigens must be present on the surfaces of dendritic (DC) or other antigen-presenting cells. To examine the effects of Sf9-rHA, Mimic-rHA, CHO-rHA and CHO-rHA(+NA) on antigen presentation, we treated bone marrow-derived DCs with LPS for 1 h, followed by incubation with four groups of rHAs for 3 h and co-culturing with splenocytes obtained from mice immunized with Sf9-rHA. Bone marrow-derived DCs pulsed with Sf9-rHA resulted in the strongest presentation of stimulated IFN-γ-secreting T cells, followed by DCs pulsed with Mimic-rHA and CHO-rHA (**Figure 6C**). CHO-rHA stimulation was slightly restored by removing the terminal sialic acids of HA—that is, CHO-rHA(+NA). Higher levels of IL-2-secreting T cells were also observed in DCs pulsed with Sf9-rHA compared to the other three groups (**Figure 6D**). Parallel studies using animals immunized with Sf9-rHA, Mimic-rHA, CHO-rHA, and CHO-rHA(+NA) reacted with DCs pulsed with each of different constructs also showed that Sf9-rHA resulted in the strongest presentation of stimulated IFN-γ-secreting and IL-2-secreting T cells, indicating that the T cells responded best to DCs pulsed with Sf9-rHA no matter which HA molecule the mice were immunized with (**Figure S1**). Combined, these results indicate a correlation between stronger T cell responses elicited by Sf9-rHA immunization and increases in antigen-presenting DCs.

### Neutralizing antibodies against homologous and heterologous H5N1 virus clades

**Figure 7:**
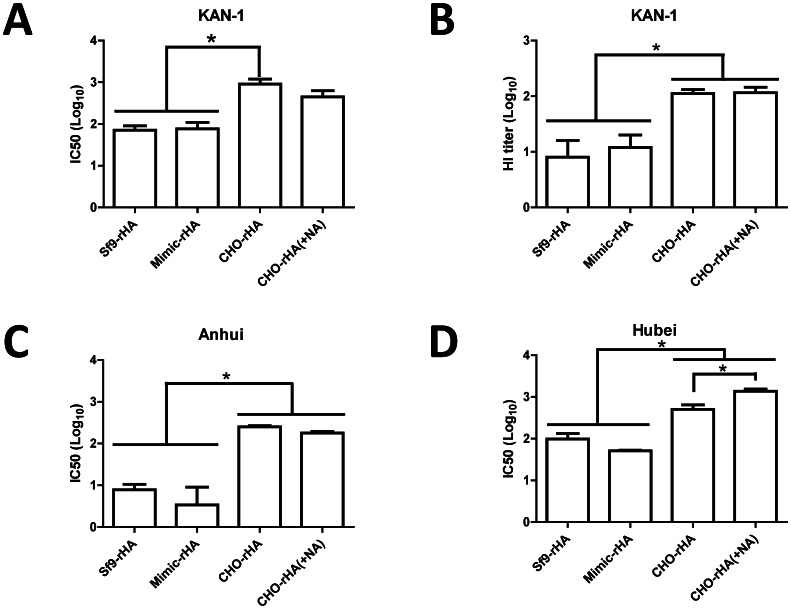
Neutralizing antibodies against homologous and heterologous clades of H5N1 viruses. (A) Neutralizing antibodies were measured as the reduced luciferase activity of H5HA-pseudotyped particles (H5pp) following the incubation of sera with homologous (KAN-1; clade 1) H5-pseudotyped particles. (B) HI antibody titers against the KAN-1 virus were determined by reacting influenza VLPs with turkey red blood cells. Cross-clade neutralizing antibodies against (C) Cclade 2.3.4 of A/Anhui/1/2005 and (D) clade 2.3.2 of A/Hubei/1/2010 were also determined using H5pp assays. Data represent mean ± standard deviation. Results were analyzed using one-way ANOVAs with Tukey's tests, with statistical significance at *p*<0.05 as asterisks indicated.

KAN-1 H5pp neutralization assays were performed with neutralizing antibody titers from antisera collected from the four immunization groups against the homologous clade 1 virus. Results indicate serum dilution-dependent neutralization curves for all four groups, but with the corresponding IC50 value from the CHO-rHA immunization group approximately one log higher than the IC50 values for the Sf9-rHA and Mimic-rHA groups (**Figure 7A**). Immunization via the removal of terminal sialic acid (complex type N-glycans) resulted in a lower but statistically insignificant value (**Figure 7A**). HI antibody titers against the KAN-1 clade virus were determined using influenza VLPs reacted with turkey red blood cells. Results indicate that the HI titers for the CHO-rHA and CHO-rHA(+NA) immunization groups were also approximately one log higher than those for the Sf9-rHA and Mimic-rHA groups (**Figure 7B**).

Since the re-emergence of H5N1 viruses in 2003, many new clades and subclades have evolved with various glycosylation patterns, consisting of six highly conserved glycosylation sites and six diverse glycosylation sites [32]. The H5N1 virus strain A/Thailand/1(KAN-1)/2004 is the first human isolate from a fatal case in Thailand and belongs to clade 1. Some clade 2.3.4 H5N1 viruses isolated from China and Vietnam, such as A/Anhui/1/2005, contain conserved glycosylation sites in HA, but other dominant viruses from clade 2.3.2, such as A/Hubei/1/2010, show the loss of glycosylation at 170N in HA, as well as most H5N1 viruses in clade 2.2 [32]. Thus, cross-clade neutralizing antibody titers were also determined against two heterologous clades: clade 2.3.4 for A/Anhui/1/2005 and clade 2.3.2 for A/Hubei/1/2010. Neutralizing IC50 titers from the CHO-rHA and CHO-rHA(+NA) immunization groups were also significantly higher than those obtained from the Sf9-rHA and Mimic-rHA groups, in both cases against Anhui H5pp (**Figure 7C**) and Hubei H5pp (**Figure 7D**).

### Protective immunity in mice following live virus challenge

**Figure 8:**
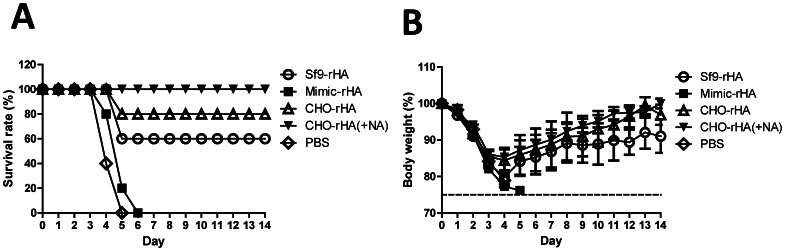
Protective immunity in mice. Female BALB/c mice (6−8 wk old, five mice per group) were intramuscularly immunized with 20 µg of recombinant HA proteins over a 3-week interval. Immunized mice were intranasally challenged with 10× MLD_50_ H5N1 virus (NIBRG-14) 3 wk after their second inoculations. Survival rates (A) and body weights (B) were recorded for 14 d. Body weight loss over 25% was used as an end-point. Body weight loss is presented as mean ± standard deviation.

We used challenges with 10×MLD_50_ H5N1 live viruses (NIBRG-14) to assess the protective immunities of mice in the four immunization groups 3 wk following their second inoculations. At a dosage of 20 µg HA proteins, survival rate (**Figure 8A**) and reduced body weight loss (**Figure 8B**) both followed a high-to-low order of CHO-rHA(+NA) > CHO-rHA > Sf9-rHA > Mimic-rHA. Immunized mice with HA expressed in CHO cells with or without NA treatment had 80−100% protection 14 days following the live virus challenges; immunized mice with HA expressed in insect cells up to 60% protection. Reduced body weight loss data indicate that HA expressed in CHO cells resulted in less body weight loss compared to HA expressed in insect cells.

## Discussion

When developing H5N1 subunit vaccines, protective immunity should correlate with the titers of HI, neutralizing, or other high-quality antibodies rather than antigen-binding antibodies. According to our present results, recombinant HA glycoproteins containing complex terminally sialylated and asialyated-galactose type N-glycans induced higher levels of HI and neutralizing antibodies and better protective immunity in mice compared to recombinant HA glycoproteins containing pauci-mannose or high-mannose type N-glycans. While the antibody titers are higher with the insect cell derived HA proteins, the neutralization titers are much higher with the mammalian cell produced HA proteins with complex type N-linked glycans and the antibodies protect mice to lethal challenge. The results are highly relevant to issues that should be considered in the production of fragment vaccines.

Our finding that CHO-rHA induced higher HI and neutralizing antibody titers than Sf9-rHA is consistent with other studies involving recombinant HA expressed in HEK293 cells [7], [8]. CHO-rHA contains bi-, tri-, and tetra-antennary complex type N-glycans with terminal sialic acid, whereas Sf9-rHA mainly consists of pauci-mannose and high-mannose type N-glycans. It was recently reported that recombinant HA proteins carrying terminal mannose residues induce lower HI antibody titers than HA proteins that carry complex glycans or single GlcNAc units [7], [17]. The findings of the present study are somewhat contradictory to the above in as much as recombinant HA proteins carrying low or high mannose glycans elicited higher titers of HA-specific antibodies and stronger T cell responses. Indeed, the higher the glycan content of tri- and tetra-antennary complex glycans, the greater was the neutralizing and HI titers and consequent protective effect. Therefore, the increased HI and neutralizing antibody titers associated with CHO-rHA are not due to suppressed DC activation by the terminal mannose moieties of HA glycoprotein, as has been reported for influenza HA [7] and HIV-1 gp120 [33]. Instead, we found that Sf9-rHA induced the highest levels of HA-specific total IgG and avidity in three of the four IgG subtypes (IgG2a, IgG2b and IgG3), as well as higher levels of IFN-γ-secreting T cells and increased DC antigen presentation efficiency. Although the purified proteins of Sf9-rHA, Mimic-rHA, and CHO-rHA have identical amino acid sequences, the antigen presentation efficiencies of these antigens were significantly different (**Figure 6C, D** and **Figure S1**). As it was known that intracellular antigen uptake, routing, and presentation are associated with distinct endocytic receptors such as C-type lectins [**34]**, [**35**], the increased antigen presentation efficiency by Sf9-rHA may be through mannose-terminated N-glycans interacted with lectin receptors guiding to early endosomal compartment but still required further investigation.

CHO-rHA and CHO-rHA(+NA) displayed lower IgG2a:IgG1 ratios compared to Sf9-rHA and Mimic-rHA (0.65 and 2.90 versus 8.00 and 7.30, respectively), suggesting that the Th2 skewing response was favored by recombinant HA proteins containing sialic acid-terminated or galactose-terminated complex type N-glycans. CHO-rHA induced higher neutralizing and HI antibody titers but not total antibody titers, indicating that higher glycan forms in the CHO-derived recombinant HA proteins improved functional antibody titers, which was not correlated with total antibody titers. Although CHO-rHA induced higher IgG1 antibody titers and neutralizing antibody titers, there were no evidences to confirm the correlation between increased IgG1 antibody titers and increased neutralizing antibody titers in this study.

Glycan patterns and topological shielding in influenza HA molecules can affect their receptor binding to sialic acid. Greater sialic acid binding has been reported following the trimming and modification of the oligosaccharides of N-glycans such as single GlcNAc [17], pauci-mannose, and high-mannose [7], [36]. Removal of the terminal sialic acids of complex type N-glycans increased sialic acid binding [8], [17], [36]. Results from fetuin binding assays with Sf9-rHA, Mimic-rHA, CHO-rHA and CHO-rHA(+NA) indicate a high-to-low binding activity order of Sf9-rHA > Mimic-rHA > CHO-rHA(+NA) > CHO-rHA (**Figure S2**). Since fetuin contains both α2-3- and α2-6-linked sialic acids, we used a 98-glycan array to analyze these recombinant HA proteins; our results indicate binding between Sf9-rHA and 18 of 26 α2-3-linked sialic acids, but not between Sf9-rHA and any of the 13 α2-6-linked sialic acids (**Figure S3**). Mimic-rHA reduced binding to α2-3-linked sialic acids by approximately 50%; no binding was observed for α2-6-linked sialic acids. Relatively low bindings were found for CHO-rHA and CHO-rHA(+NA) to α2-3-linked sialic acids, as compared to strong binding to Sf9-rHA. The binding intensity of CHO-rHA to α2-3-linked sialic acids decreased by almost 25 folds as compared to Sf9-rHA; however, CHO-rHA(+NA) further increased the binding to α2-3-linked sialic acids by approximate 3 folds. In contrast, immunization with CHO-rHA elicited higher levels of HI and neutralizing antibodies, and better protective immunity against challenges with live viruses. These results conflict with a recent description of single GlcNAc HA N-glycans increasing the receptor binding of sialic acids, as well as correlations between this increase and increases in both neutralizing antibody titers and protective immunity in mice [17]. Accordingly, recombinant HA protein antigenicity and immunogenicity may not be directly associated with the receptor binding activity of α2-3-linked sialic acids.

Our results indicate that the removal of terminal sialic acid from bi-, tri-, and tetra-antennary HA complex-type N-glycans improved protective immunity, as determined by reductions in body weight loss (CHO-rHA(+NA) in **Figure 8B**). The enhanced protective immunity provided by the removal of sialic acid by neuraminidase can be also due to CHO-rHA protein degradation induced by 37°C/6hr treatment which requires further confirmation. We also found that the terminal sialic acids of complex-type HA glycoproteins did not significantly affect neutralizing antibody titers against homologous and heterologous H5N1 virus clades (**Figure 7**), but did induce higher levels of IFN-γ-secreting T cells and increase DC-associated antigen presentation efficiency (**Figure 6**). It was reported that recombinant HA proteins from Sf9 and HEK293 cells to show that anti-H5N1 neutralizing antibody responses are not dependent on terminally sialyated N-glycans [8]. Increased DC binding has been reported following the removal of HIV-1 gp120 terminal sialic acid [37], therefore improved protective immunity from galactose-terminated complex N-glycans may also be due to increased interaction with DCs. In a separate study, HA N-glycans of the PR/8/34 (H1N1) influenza A virus produced in MDCK and Vero cells were all asialyated, with the former containing larger, tri- or tetra-antennary, alpha- and beta-galactose-terminated N-glycans, and the latter containing smaller, exclusively beta-galactose-terminated N-glycans [38]. MDCK cell-derived viruses with the N-glycan pattern have been found to favor antibody responses, while Vero cell-derived influenza viruses with the N-glycan pattern have been described as increasing differential T cell responses mediated by DCs [39]. In the present study, immunization with Mimic-rHA (primarily containing galactose-terminated, bi-antennary, and high-mannose type complex N-glycans) yielded lower levels of protective immunity and decreases in body weight loss (**Figure 8**). This suggests that tri- or tetra-antennary complex-type N-glycans are required for inducing protective immunity against H5N1 virus infection. This strongly suggests that recombinant HA glycoproteins with tri- or tetra-antennary complex type N-glycans (obtainable from engineered CHO cells) have potential for use in subunit vaccine development. Since ferrets are regarded as the most appropriate animal model for H5N1 pathogenesis and therapeutic and prophylactic development [3], [40], [41], the differences and similarities in the mouse, ferret, and human immune response and the projected testing of these recombinant HA vaccines require further investigation.

## Materials and Methods

### Ethics Statement

The animal studies were conducted in accordance with guidelines established by the Laboratory Animal Center of National Tsing Hua University (NTHU). Animal use protocols were reviewed and approved by the NTHU Institutional Animal Care and Use Committee (approval no. 09931). Mouse challenge experiments were evaluated and approved by the Institutional Animal Care and Use Committee of Academia Sinica.

### Recombinant HA protein expression and purification in insect cells

The soluble recombinant HA-expressing coding sequences in the three cell lines included the HA ectodomain of the A/Thailand/1(KAN-1)/2004 (H5N1) strain followed by a GCN4pII termerization motif and a His tag as described in Lin et al. (2011) [28]. Sf9 cells (ATCC CRL-1711) were grown in SF900-II serum-free medium (Invitrogen) at a density of 2×10^6^ cells/ml and infected with recombinant baculoviruses produced by the Bac-to-Bac expression system (all Invitrogen). Mimic cells (Invitrogen) were grown in SF900-II medium supplemented with 5% FBS and infected with recombinant baculoviruses. After 2 d post-infection, supernatants were collected for HA purification using nickel-chelated affinity chromatography (Tosoh).

### Recombinant HA protein expression and purification in CHO cells

The vector expressing HA proteins was a modified pISID vector [19]. Following transfection, CHO/dhfr- cells (ATCC CRL-9096) were selected using MEMα without RNS/dRNS supplemented with 10% dialyzed FBS and 200 µg/ml Zeocin (Invitrogen). High-producing stable CHO cell clones were selected after screening from 80-100 transfected stable clones in CHO/dhfr^−^ cells with stepwise increases in MTX (Sigma) concentration from 0.02 to 1.00 µM. Culture supernatants of the amplified cell clones were collected for CHO-rHA protein purification as described above. For CHO-rHA(+NA) preparation, purified CHO-rHA was incubated with excessive neuraminidase from *Vibrio cholerae* (100 mU/mg rHA) at 37°C for 6 h to ensure the complete cleavage. The recombinant proteins of CHO-rHA and CHO-rHA(+NA) were purified using nickel-chelated affinity chromatography.

### Glycan analysis

Purified recombinant HA proteins were placed on SDS-PAGE gels and the corresponding band was cut out of the gel. The N-linked glycans of the proteins were released following the protocol described previously [24]. Briefly, the cut gel was frozen at −20°C for 2 h and then sliced in 1 mm^2^ pieces. The pieces were washed twice with 20 mM NaHCO_3_ (pH 7.0). The washes were discarded and replaced with 1∶1 acetonitrile: 20 mM NaHCO3. After incubation for 60 min, the gel pieces were completely dried and then deglycosylated with PNGase F (Roche) overnight at 37°C. Glycans were isolated from protein backbones using 10 kDa MWCO filters, dried in a SpeedVac centrifuge, and labeled with 2-aminobenzamide (2-AB). After removing excess 2-AB, HILIC-HPLC, an alternative HPLC for polar compound separation [42], was used to separate individual glycan structures, and labeled glycan samples were exposed to 4 exoglycosidase enzymes (sialidase, galactosidase, hexosaminidase and fucosidase, all Prozyme) to cleave specific sugar molecules prior to another round of HILIC-HPLC. A dextran ladder generated by HILIC-HPLC was used as a standard to obtain glucose unit (GU) values for individual peaks recognized in glycans from rHA antigens. The elution times obtained for the different glycan peaks were converted to GU by comparison with the elution of fractions from a dextran ladder as reported previously [21].

### Mouse immunization

Female BALB/c mice (6−8 wk old, five mice per group) were immunized with 20 µg of recombinant HA proteins mixed with 10% PELC and 10 µg CpG [27], [28]. Intramuscular injections were given during weeks 0 and 3 and blood collected in week 5. Spleens were collected and splenocytes isolated 1 d after second bleedings.

### ELISA assay

Individual wells in 96-well plates were coated with recombinant HA proteins (2 µg/ml) expressed in Sf9 and CHO cells (100 µl/well) and blocked with 1% BSA. Antibody isotypes and subclasses were detected with ELISAs using goat anti-mouse IgM, IgA, IgG, IgG1, IgG2a, IgG2b and IgG3 (Bethyl Laboratories, Inc.). For avidity ELISA assays, plates were incubated with serum samples for 1 h at 37°C prior to the addition of 6 M urea and another incubation for 5 min at 37°C followed by 3 washes as described previously [29]. Endpoint titers were calculated as the most dilute serum concentrations giving optical density readings >0.2 above a negative control. IgG avidity was calculated as end point titer with urea/end point titer without urea x 100.

### Splenocytes and bone marrow-derived DCs preparation

For splenocytes isolation, spleen was removed from mouse and mashed through 70 µm cell strainer to release cells. Cells were spun down and re-suspended in 5 ml ACK lysis buffer (Invitrogen) for 5 min at RT. After cells spun down, dead cell mass in supernatant was removed; splenocytes (>80% CD3^+^ cells) were re-suspended and used for all experiments.

Bone Marrow cells were isolated from femurs and tibias and seeded in 24-well plate with 1 ml supplemented RPMI-1640 medium (10% FBS, 2 mM L-glutamine, nonessential amino acids, sodium pyruvate, HEPES) (all from Invitrogen), 0.5 mM 2-ME (Sigma-Aldrich), and 15 ng/ml recombinant mouse GM-CSF (Invitrogen). On day 3, 1 ml of medium containing 15 ng/ml GM-CSF was added, and half the cell-free supernatant was exchanged by fresh medium containing 10 ng/ml GM-CSF on day 5. The 7-day-cultured bone marrow-derived DCs (>70% CD11c^+^ cells) were used for all experiments.

### Detection of HA-specific antibodies secreting B cells in spleens

Multiscreen 96-well filtration plates (Millipore) were coated with recombinant HA expressed in Sf9 and CHO cells and blocked with 10% fetal bovine serum in RPMI. Freshly prepared splenocytes were added to each well (5×10^5^ cells/well) and incubated for 2 d at 37°C with 5% CO_2_. Detection was performed by ELISPOT using HRP-conjugated anti-mouse IgG antibodies and an AEC staining kit (Sigma). Spot counting was performed using a Cellular Technology ImmunoSpot ELISpot reader.

### Detection of HA-specific cytokine-secreting T cells in spleens

IFN-γ- and IL-4-secreting T cells were determined by ELISPOT assays using Ready-Set-Go IFN-γ and IL-4 kits (eBioscience). Following the manufacturer’s instructions, anti-mouse IFN-γ and IL-4 antibodies were used to coat multiscreen 96-well filtration plates that were blocked with 10% fetal bovine serum in RPMI. Freshly prepared splenocytes were added to each well (5×10^5^ cells/well) and stimulated with 1 µg/ml pooled peptides (15-mer overlapped by 8 amino acids) spanning the HA_1_ of H5HA (A/Viet Nam/1203/2004). Plates were incubated for 2 d at 37°C with 5% CO_2_. Spot development and counting was conducted as described above.

### Antigen presentation assay

Dendritic cells (DCs) derived from bone marrow were matured with 10 µg/ml LPS for 1 h and loaded with 40 µg/ml recombinant HA proteins for 3 h followed by 3 washes. Cells were fixed with 1% paraformaldehyde for 15 min followed by quenching with 0.5 M glycine for 15 min. Next, treated cells (1×10^6^ cells/ml) were co-incubated with 5×10^6^ splenocyte cells/ml collected from mice immunized with Sf9-rHA, Mimic-rHA, CHO-rHA, or CHO-rHA(+NA) for 2 d. Antigen presentation was determined by measuring IFN-γ- and IL-2-secreting T cells using Ready-Set-Go IFN-γ and IL-2 kits (eBioscience) as described above.

### HI assay

Sera were treated with receptor-destroying enzyme (Denka Seiken) for 18 h at 37°C followed by an additional treatment at 56°C for 30 min. Treated sera were serially diluted two-fold (starting from 1∶10) and incubated with 4 HA units of A/Thailand/1(KAN-1)/2004 VLPs as we previously reported [43] for 30 min at room temperature. Next, 0.5% turkey RBCs were added prior to another incubation for 30 min at room temperature. HI titers were determined as the reciprocal of the highest dilution completely inhibiting hemagglutination.

### H5 pseudotyped particle (H5pp) neutralization assay

HEK293A cells (Invitrogen) were co-transfected with pNL-Luc-E^−^ R^−^ pcDNA3.1(+) expressing the HAs of A/Thailand/1(KAN-1)/2004 (clade 1), A/Anhui/1/2005 (clade 2.3.4), or A/Hubei/1/2010 (clade 2.3.2) strains, and pcDNA4B expressing the NA of the A/Viet Nam/1203/2004 strain. Culture supernatants were collected and concentrated 48 h post-transfection. Neutralizing antibodies were quantified as the reduced luciferase expression level following H5pp transduction in MDCK cells as described previously [28], [44]. Briefly, neutralizing antibodies were quantified as the reduced luciferase expression level following H5pp transduction in MDCK cells. H5pp (50 µl) (50TCID_50_) was incubated with 50 µl of a two-fold serial dilution of anti-sera (starting dilution 1∶20) for 1 h at 37°C prior to the addition of MDCK cells (1.5×10^4^ cells/well). Culture medium was removed 2 d post-infection and cells were lysed using Glo Lysis Buffer (Promega). Luciferase activity was measured by the addition of neolite luciferase substrate (PerkinElmer). Neutralization titers (IC50) represent the serum dilution required to obtain a 50% reduction in RLU compared to control wells with the virus alone.

### Viral challenge

BALB/c mice (6-8 wk old, five mice per group) were inoculated with two doses of recombinant HA vaccine (20 µg/dose); PBS-immunized mice were used as a mock control. To investigate protective immunity, immunized mice were intranasally challenged with a lethal dose (10×MLD_50_) of reassortant H5N1 influenza virus (NIBRG-14) at 3 wk following the second immunization. Mouse survival rates and weight losses were monitored daily for 14 d. According to IACUC guidelines, body weight loss over 25% was used as an end-point.

### Fetuin binding assays

96-well plates were coated with 100 µg/ml fetuin overnight at 4°C, and then blocked with 1% BSA in PBS buffer followed by washing three times with 0.05% Tween 20/PBS buffer. Serially diluted rHA proteins were pre-complexed with HRP-conjugated anti-His tag antibodies (Bethyl Laboratories, Inc.) for 30 min and then added to individual plates to incubate for 60 min at room temperature. After three washes, HA binding was detected using ELISAs (450 nm OD).

### Glycan Microarray Fabrication

Microarrays were printed (BioDot; Cartesian Technologies) by robotic pin (SMP3; TeleChem International) deposition of 0.7 nL of various concentrations of amine-containing glycans in printing buffer (300 mM phosphate buffer with 0.005% Tween 20; pH 8.5) from 96-well plates onto NHS-coated glass slides (NexterionHslide; SCHOTT North America). Slides were spotted with each 50 µM glycan solution and designed to hold 14 grids. Printed slides were allowed to react in 80% humidity for 1 h followed by overnight desiccation and storage in a desiccator at room temperature until use. Before binding assays were performed, slides were blocked with 50 mM ethanolamine in borate buffer (pH 9.2) and washed twice with water and PBS buffer (pH 7.4).

### Glycan array analyses

Recombinant HA proteins dissolved in wash buffer (0.005% Tween 20/PBS buffer, pH 7.4) were added to cover glycan array grids with coverslips. After incubation in a humidified chamber for 1 h, slides were washed three times with wash buffer. Next, rabbit anti-H5N1 HA antibodies were added to each slide, followed by another 1 h of incubation in a humidified chamber. After another three washes, Cy3-conjugated goat anti-rabbit IgG antibodies were added to each slide, followed by incubation in a humidified chamber for 1 h. Slides were again washed three times with wash buffer, PBS buffer, and H_2_O before drying. Slides were scanned at 595 nm (for Cy3) with a microarray fluorescence chip reader (GenePix Pro 6.0; Molecular Devices).

### Statistical analysis

All results were analyzed using two-tailed Student’s *t* tests or one-way ANOVAs with Tukey's tests, with statistical significance at *p*<0.05. All experiments were performed at least twice, with similar results.

## Supporting Information

Figure S1 **T-cell stimulation by antigen-presenting dendritic cells.** Pre-treated DCs with LPS and recombinant HA proteins were co-incubated with splenocytes from mice immunized with different recombinant HA proteins for 2 d. Antigen presentation was determined by measuring (A) IFN-γ- and (B) IL-2-secreting T cells using ELISPOT assays. DCs pretreated with LPS and pulsed with PBS were used as a negative control. Data represent mean ± standard deviation.
(TIFF)

Figure S2 **Fetuin binding assays.** 96-well plates were coated with 100 µg/ml fetuin and incubated with serially diluted recombinant HA pre-complexed with HRP-linked anti-His tag antibodies for 60 min at room temperature. HA binding was subsequently detected by ELISAs (450 nm OD). Data represent mean ± standard deviation.
(TIFF)

Figure S3 **Glycan array analyses.** Recombinant HA proteins were added to a 98-glycan array and incubated for 1 h at 37°C. Rabbit anti-H5HA antibodies were added and incubated for 1 h. Cy3-conjugated goat anti-rabbit IgG antibodies were added next, followed by incubation for another 1 h. Binding activity is shown as fluorescence intensity detected by scanning with a microarray fluorescence chip reader set at 595 nm (for Cy3). Black arrows: 18 of 26 α2-3 linked sialic acids bound with Sf9-rHA; gray arrows: 0 of 13 α2-6 linked sialic acids bound with Sf9-rHA.
(TIFF)
