# Supplementary figures and images for: Different Immunity Elicited by Recombinant H5N1 Hemagglutinin Proteins Containing Pauci-Mannose, High-Mannose, or Complex Type N-Glycans
Source: PLoS One. 2013 Jun 14;8(6):e66719. doi: 10.1371/journal.pone.0066719 (PMC3682957; doi:10.1371/journal.pone.0066719)

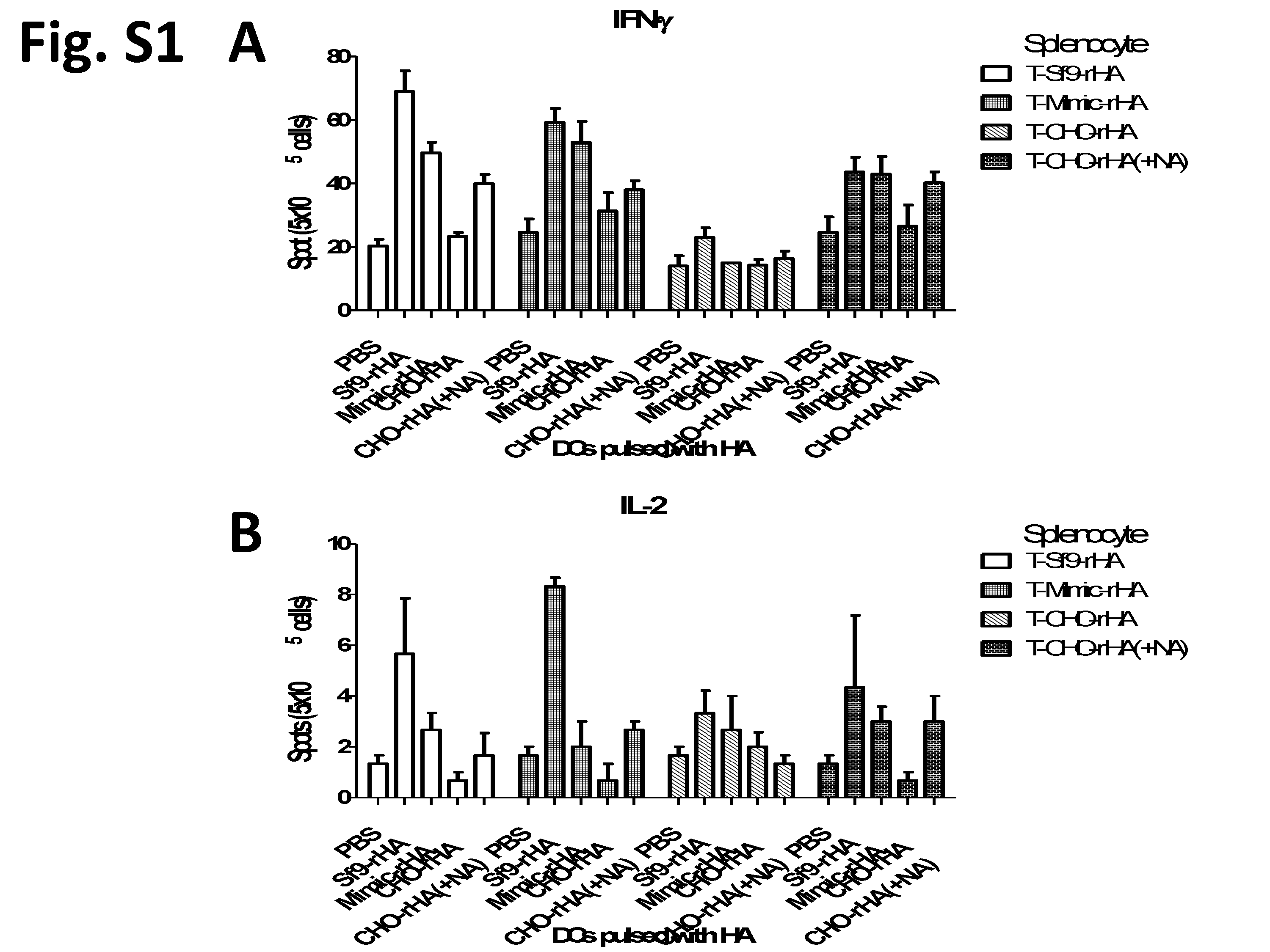

Supplement: Figure S1 — T-cell stimulation by antigen-presenting dendritic cells. Pre-treated DCs with LPS and recombinant HA proteins were co-incubated with splenocytes from mice immunized with different recombinant HA proteins for 2 d. Antigen presentation was determined by measuring (A) IFN-γ- and (B) IL-2-secreting T cells using ELISPOT assays. DCs pretreated with LPS and pulsed with PBS were used as a negative control. Data represent mean ± standard deviation. (TIFF) [file pone.0066719.s001.tiff]

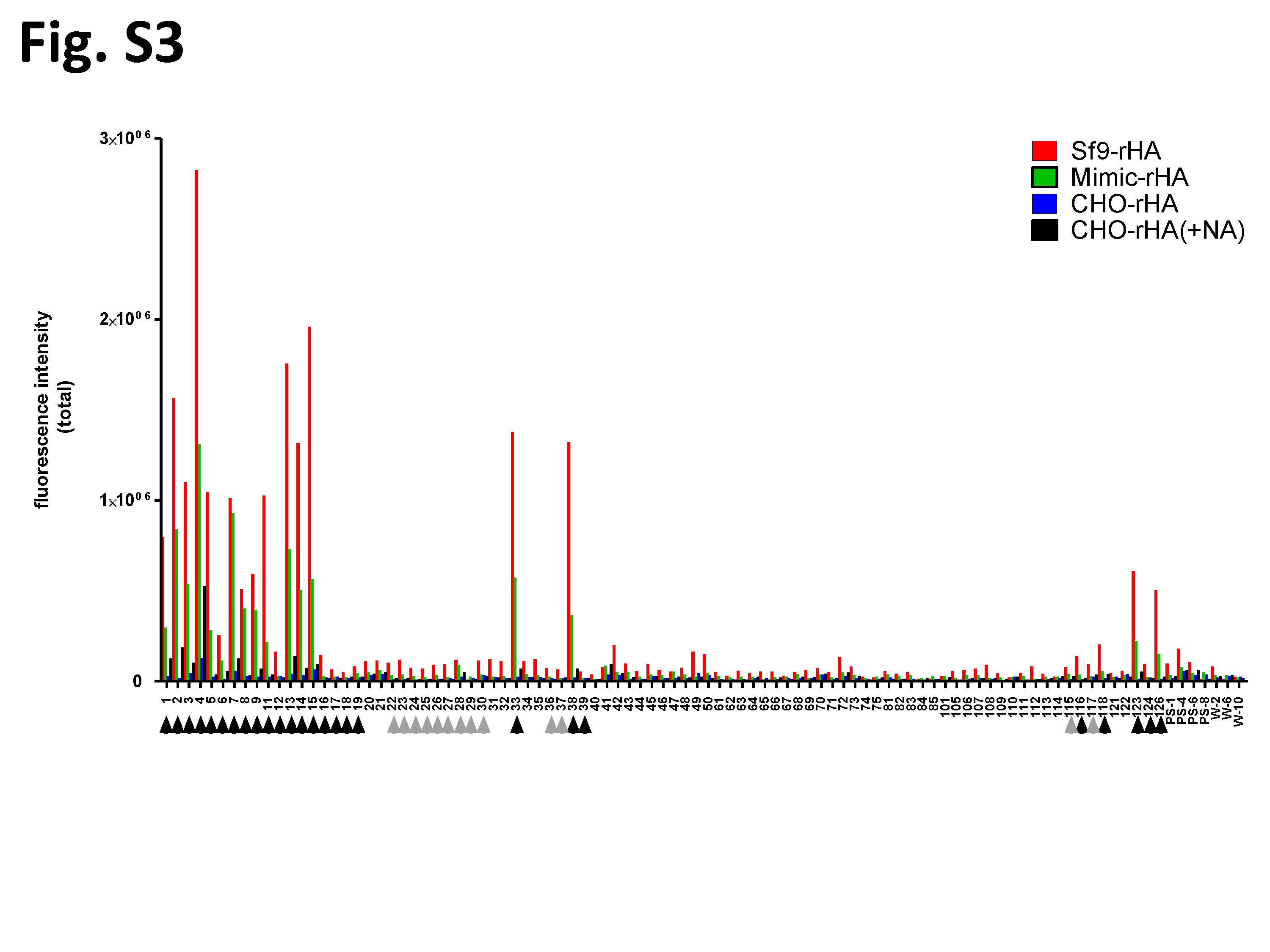

Supplement: Figure S3 — Glycan array analyses. Recombinant HA proteins were added to a 98-glycan array and incubated for 1 h at 37°C. Rabbit anti-H5HA antibodies were added and incubated for 1 h. Cy3-conjugated goat anti-rabbit IgG antibodies were added next, followed by incubation for another 1 h. Binding activity is shown as fluorescence intensity detected by scanning with a microarray fluorescence chip reader set at 595 nm (for Cy3). Black arrows: 18 of 26 α2-3 linked sialic acids bound with Sf9-rHA; gray arrows: 0 of 13 α2-6 linked sialic acids bound with Sf9-rHA. (TIFF) [file pone.0066719.s003.tiff]
